# Toward Reliable Estimates of Abundance: Comparing Index Methods to Assess the Abundance of a Mammalian Predator

**DOI:** 10.1371/journal.pone.0094537

**Published:** 2014-04-17

**Authors:** Denise Güthlin, Ilse Storch, Helmut Küchenhoff

**Affiliations:** 1 Chair of Wildlife Ecology and Management, University of Freiburg, Freiburg, Baden-Württemberg, Germany; 2 Statistical Consulting Unit, Department of Statistics, University of Munich, Munich, Bavaria, Germany; Université de Sherbrooke, Canada

## Abstract

Due to time and financial constraints indices are often used to obtain landscape-scale estimates of relative species abundance. Using two different field methods and comparing the results can help to detect possible bias or a non monotonic relationship between the index and the true abundance, providing more reliable results. We used data obtained from camera traps and feces counts to independently estimate relative abundance of red foxes in the Black Forest, a forested landscape in southern Germany. Applying negative binomial regression models, we identified landscape parameters that influence red fox abundance, which we then used to predict relative red fox abundance. We compared the estimated regression coefficients of the landscape parameters and the predicted abundance of the two methods. Further, we compared the costs and the precision of the two field methods. The predicted relative abundances were similar between the two methods, suggesting that the two indices were closely related to the true abundance of red foxes. For both methods, landscape diversity and edge density best described differences in the indices and had positive estimated effects on the relative fox abundance. In our study the costs of each method were of similar magnitude, but the sample size obtained from the feces counts (262 transects) was larger than the camera trap sample size (88 camera locations). The precision of the camera traps was lower than the precision of the feces counts. The approach we applied can be used as a framework to compare and combine the results of two or more different field methods to estimate abundance and by this enhance the reliability of the result.

## Introduction

Reliable knowledge on the abundance of a species is desirable for managers when establishing wildlife management practices [1], [2]. However, there are only few species and landscapes that allow absolute abundance to be obtained on a large scale with the prevailing time and cost constraints most studies are under. On the other hand, for most decisions on suitable conservation practices, knowledge of the relative abundance is sufficient. Further, information on factors influencing species abundance, such as landscape variables, can be derived without knowledge of absolute abundance.

Indices are a cost effective way to estimate the relative abundance of a species [3]. There is a large variety of field methods to choose from for obtaining an index of abundance, whereas the most appropriate field method to be used depends on the species of interest and the landscape where the research is taking place [4]. Commonly, abundance indices are based on animal signs (i.e. track, vocal, den or feces counts), photographs obtained with remote camera traps, and records of hunting bags or road traffic casualties.

One major challenge with indices of abundance is that they are prone to various sources of bias, for example due to differences in the persistence or detectability of signs among seasons or habitats. To reduce possible sources of bias a standardized sampling protocol is required. Where this is not feasible, possible sources of bias can be included as covariates in a regression framework [5]. Still, some sources of bias may remain undetected. Another challenge is the unknown relationship between the index values and true abundance; it might not be linear and at worst not even monotonic. Using and comparing two or more dissimilar field techniques, can enable researchers to detect discrepancies that are caused by bias or a non monotonic relationship.

There is a vast amount of studies that compare different index methods with each other or with estimates of absolute abundance. Most studies only compare the index values obtained in terms of detection efficiency, precision and/or correlation (e.g. [1], [4], [6]–[12]). None of these studies, however, used a framework that includes possible sources of bias as covariates in a regression model. Regression models can easily be used to include variables possibly biasing an index (e.g. [13]). Further, they can be expanded to extract information on factors that might influence species relative abundance such as landscape variables (e.g. [14]). By comparing the effects of these factors, such an approach also enables comparison of indices not gathered at exactly the same locations. To our knowledge the approach of inclusion of possible sources of bias in a regression analysis for obtaining unbiased estimates of the variables of interest has not been used in the abundance estimation of mammalian carnivores so far. On the other hand, this approach is often used in other fields and is an appealing advantage of multiple regression models.

Usage of automatic cameras has become very popular during the last years in wildlife research, especially if the animals under investigation are cryptic or elusive. Automatic cameras facilitate species inventory (e.g. [15]–[17]) and allow abundance estimates of species that can be individually identified from pictures using capture-recapture techniques (e.g. [18]–[20]). For species that cannot be individually identified, photographic rate has been used as an index of abundance (e.g. [21]–[23]). A close correlation between the index derived from cameras with absolute abundance has been shown for tigers (*Panthera tigris*; [21]) and Harvey's duikers (*Cephalophus harveyi*; [22]). [24] suspects fewer sources of bias for indices derived from camera traps than from other field methods.

Before the rise of camera traps, feces, pellet or dung counts maybe have been the widest used method to derive indices of abundance, as they are practical for many cryptic species in different habitats and are cost effective. While new camera technology has eagerly been adopted by many scientists, it remained unclear whether camera traps really are better than conventional feces counts in terms of effectiveness and efficiency.

In this study, we applied two independently collected indices of red fox (*Vulpes vulpes*) abundance to obtain reliable estimates at the landscape scale: the frequency of feces along line transects and the frequency of photographs obtained by camera traps. We used negative binomial regression models, to independently identify landscape parameters that influence the feces frequency or the photographic rate, respectively. In these models, we included factors that we assume to possibly bias the index methods. Then, we used the obtained regression coefficients to predict and map relative abundance. We compared fecal-based and camera-based model coefficients and model predictions. Further, we compared the economical costs and the precision of the two methods. Our objectives were to provide and use a framework, that allows the comparison of two or more different abundance estimates and to provide information on the costs and precision of feces counts and camera traps to support researchers and managers in choosing appropriate field techniques. This paper builds on a previous study, in which we employed regression models to identify landscape parameters influencing red fox abundance based on feces counts [25].

## Methods

### Ethics Statement

We obtained the permission to perform line transect searches within nature reserves from the Environmental Department at the Regierungspräsidium Freiburg. For all other areas no permission was required as unrestricted rights to access nature apply. We located all cameras in state owned forests of five counties. We obtained the permission for this from the Landratsämtern of the five counties: Emmendingen, Rastatt, Calw, Waldshut-Tiengen and Breisgau-Hochschwarzwald. Both field methods were indirect methods and did not involve direct encounter with animals.

### Study area

This study was carried out in the Black Forest, a low mountain range located in south-western Germany, which ranges from 120 to 1493 m a.s.l. Two thirds of the Black Forest's approximately 6000 km^2^ are forested. The annual mean temperature ranges from between 4°C at higher elevations and 10.4°C in the valleys [26]. Forests are conifer-dominated. Landscape composition varies significantly across the Black Forest. Large continuous forest dominates the northern uplands, whereas in the southern uplands forest is intermixed with grasslands and settlements; in the valleys and in the eastern part of the Black Forest mosaics of forest, grassland, agricultural fields and settlements dominate the landscape.

### Study design

#### Feces counts

**Figure 1:**
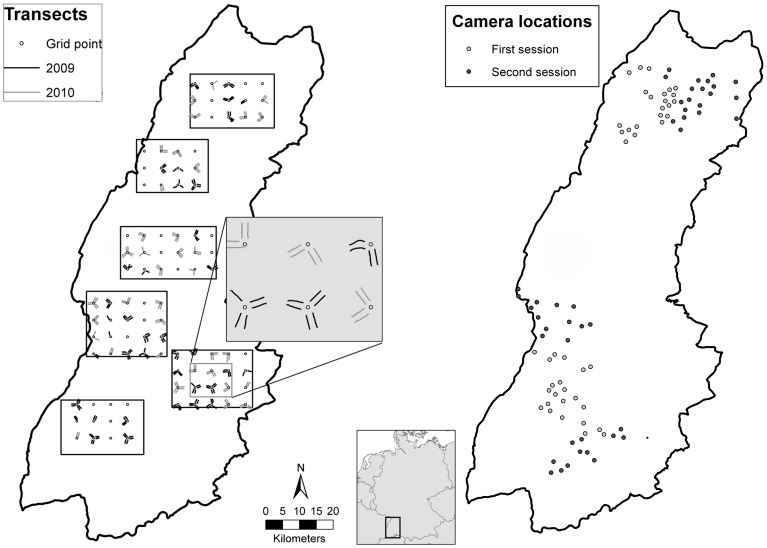
Map of the Black Forest. Transects of the feces counts (left): black rectangles indicate study rectangles, lines indicate transects searched (black: 2009, grey: 2010). Locations of the camera traps (right): grey circles indicate location of camera traps of the first session from 16.04. to 11.05.2012, black circles indicate locations of the second session from 24.05. till 15.06.2012. The left figure is reprinted from [25] under a CC BY license, with permission from Springer, original copyright 2013.

We used feces count data as described, analyzed, and discussed in [25]. Here, we give a short summary of the methods and refer the reader to [25] for a more detailed description. We searched for feces on line transects (length: 1.2 km), as selectively searching only along roads, tracks or linear features might be biased and is less precise than searching along line transects [27]. On a map we placed 6 study rectangles (ranging from 334 to 461 km^2^; total area: 2430 km^2^) dispersed across the Black Forest to capture most of its ecological gradient. On top, we placed a regular grid with 5 km spacing and investigated line transects at the grid points that fell within one of the study rectangles (Fig. 1). We performed line transect searches between October and the beginning of December in 2009 (134 line transects) and 2010 (132 line transects).

We searched intensively for red fox feces one meter to each side of the line transect at an average walking speed of 1 km per hour, but also recorded feces further away from the transect line. We identified feces according to their size, shape, odor and content [28]. Using an upright standing checker board (1×1 m), we recorded visibility every 300 m as the percentage of the board that was visible. We had to exclude 4 line transects from the analysis because large parts of these transects were covered by new foliage.

#### Remote camera traps

We placed 43 heat-motion triggered cameras (Cuddyback Caputre 1125, Non Typical Inc., Green Bay, WI) in the northern part of the Black Forest and 48 and in southern part (Fig.1), in two three week sessions between mid April and mid June. A pilot study had revealed that camera traps placed randomly in the forest did not yield enough red fox photographs for analysis (unpublished data); we therefore placed cameras along trails or unpaved forestry roads about 20 cm above the ground on trees. Camera locations were spaced at least 2 km apart, to minimize the chance of photographing the same individual at two different locations (compare [29]). We used the information gained from the feces counts and selected camera locations to cover the range of predicted relative abundances. We especially attempted to sample locations at high and low predicted relative abundances, as this can increase the precision of the estimation of a linear predictor (as the slope of the regression line depends much on the measurements at extreme values). Red foxes supposedly use trails more frequently in steep as compared to flat terrain [27]. We therefore selected camera locations to include steep and flat terrain across all levels of the predicted relative abundance from the feces counts.

We recorded camera locations using a global positioning system (GPS). We assigned the type of trail a camera was aimed at to one of four categories (1: animal trail, or old, out-of-use machinery road, 2: machinery road, 3: hiking trail, 4: unpaved forestry road). Further, we recorded the percentage of the ground cover in a circle with radius 3 m around the midpoint of the camera's aim, excluding the trail itself. From this we classified two categories: low ground cover (with ground cover in the circle of less or equal to one third) and high ground cover (with more than one third of ground cover in the circle).

Of the 91 different camera locations from the two trapping sessions, 88 provided pictures for further analysis. The three missing camera locations were due to theft and to camera and memory card malfunction. For all camera locations, we noted the number of red fox pictures and the number of nights the camera was operating, normally between 20 and 24 nights depending on the time of set-up and removal of camera traps. Not all camera traps were properly operating the entire session. If the time at which the camera trap ceased operating properly was evident from the recorded photographs, we used the number of trap nights the camera was operating properly. In cases where the time point was not clear, we used the midpoint between the last proper photograph and the detection of malfunction as the end of the operating time and noted the resulting trap nights. We used the approach of [23] to get an index of abundance out of all pictures obtained with the camera traps.

### Identification of landscape variables that influence relative abundance

As described in [25], we used negative binomial regression models to extract the landscape variables that influenced relative feces abundance. For the index obtained from the camera traps, we used exactly the same approach. We used the index obtained at each camera location as the dependent variable in a negative binomial regression model and included the landscape variables and possible confounding variables as explanatory variables and the log-transformed number of camera nights as the offset.

We extracted the landscape variables for each line transect and each camera location to identify the variables that influence relative feces abundance or photographic rates in a geographical information system (GIS). We placed a 1000 m buffer around each transect and camera location and extracted the mean of two metrics of primary productivity and two metrics of landscape heterogeneity for each buffer. As metrics for primary productivity we used soil quality (an index of soil texture, soil type, humus type, nutrient status and hydrological regime, compare [30]) and duration of the growing season (i.e., days above 10°C). To measure landscape heterogeneity we used landscape diversity (i.e. the Shannon Eveness Index [31]) and edge density (length of edges between different land cover types in kilometer per square kilometer).

We used the shape data provided in the German Authoritative Topographic-Cartographic Information System (ATKIS) with the program ArcGIS 10 (ESRI, Environmental Systems Research Institute, Inc., Redlands, California, USA) to extract the landscape data. We recoded the soil quality index of [30] to obtain an index with increasing values for increasing soil quality by multiplying it with minus one.

We adjusted for possible bias of the index counts, by including possible confounder variables in the regression models. In the feces count model, we included the percentage of grassland in a 2 m buffer around the line transect (due to the expected higher red fox activity, and thus marking frequency, in grassland, which is a preferred feeding habitat of red foxes [32] and to represent visibility), the slope along the line transect (as walking for the observer is more difficult in steep terrain, detection probability might be negatively affected by slope) and the mean ground visibility recorded during field work. In the camera trap model, instead, we included the slope in the 50 m buffer around the camera location (as we hypothesized the higher usage of trails as the terrain becomes steeper), the vegetation cover around the camera location (as we expected foxes to use trails more in areas with dense surrounding ground vegetation) and the type of trail the camera was located on (due to possible differences in the usage by foxes dependent on the type of trail).

In the feces count model, we included random effects for the study rectangle, grid point (to account for the nested design and possible spatial autocorrelation) and the observer (to account for individual differences in the detection probability of feces). For both index methods, we used model selection based on AIC_c_ to identify the models that were most supported by the data. We included all confounding variables and random effects in all models and only selected the landscape variables. From all models with Δ AIC_c_ <2 we calculated an averaged model and relative variable importance for each index method [33]. We calculated relative variable importance for variable *k* as the sum of the AIC weights across all the models in the set where the variable *k* occurred [33]. We used full model averaged coefficients, but reported subset averaged p-values, as the calculation of variances and p-values for full model averaged coefficients is an unresolved issue [34].

Using smooth and quadratic functions in the full model, we did not find any indication of nonlinearity of effects. Further, we did not find any indication of spatial autocorrelation in the full and the top ranked models using a permutation based Moran's I correlogram [35]. We used the statistical software package R [36] for all analyses, with the glmmADMB-glmmadmb, MuMin-dredge, ncf-mantel.test and mgvc-gam functions and used the natural logarithm as link function in the negative binomial models.

### Prediction of relative abundance

We used the results from the negative binomial models to predict and map relative fox abundance to the extent of the Black Forest separately for each of the two index methods. First, we calculated the mean value of the landscape variables in a circle with a radius of 1000 m for each cell (cell size 50 m). These values we then inserted into the negative binomial model equation of each of the best ranked models (Δ AIC_c_ <2) to obtain the predicted relative abundance for each cell. Then, we averaged the predictions for each of the two index methods using the Akaike weights. More details on the calculation of the prediction and the implementation in ArcGIS are given in [25]. Additionally, we rescaled both predictions to range between zero and one, by subtraction of the minimum and division by the range of each the methods prediction.

### Comparison of the two index methods

#### Comparison of predicted relative abundance

To compare the prediction of relative abundance of the two methods we subtracted the prediction from the camera traps of the prediction of the feces counts. Further, we calculated the Pearson correlation between the two methods' prediction of 10,000 random points, generated in ArcGIS with at least 200 m between points, to prevent clustering.

#### Comparison of costs

For the estimation of the economic cost of the two methods we separated four different categories: initial costs, running costs for equipment, travelling costs and person days, and used average cost levels in Germany in 2013. The initial cost for the feces counts consisted of three compasses, three GPS units with rechargeable batteries and charger and the materials to make the checker boards. The initial cost for the camera traps included one GPS unit with rechargeable batteries and charger, 46 camera trap units with memory cards and steel cables with pad locks to secure the camera traps on the trees. There were no running equipment costs for the feces counts, whereas the running equipment costs for the camera traps came only from batteries. Travelling costs were calculated as the driven kilometers times the kilometer rate reimbursed by Freiburg University (0.25 Euros per km). We added all hours spent for preparation, field work and data management and divided by eight to get the number of person days. We differentiated between hours worked by a qualified worker and hours worked by untrained workers. Sign surveys, such as the feces counts, depend strongly on correct identification. Training of untrained workers took between 3–5 days dependent on the worker's prior knowledge. During training the trainee accompanied a qualified worker during the feces counts. For the feces counts the person days included training time, if needed, travelling time, time for fieldwork and time for preparation and documentation. Camera trap person days included time spent on camera trap testing, camera location selection, equipment preparation and the time required to inform land owners, travel, set up and take down camera traps and screen pictures. We used the number of person days as the main unit of comparison, as the costs of labor vary significantly across the world, but we also calculated labor costs in Euro associated with our studies. The untrained workers were all university students, who completed their theses using data from the study or as part of their required course work and therefore were unpaid. To obtain the monetary value of the labor, we multiplied the days worked by qualified workers by 240 euro, the average costs for the Freiburg University for an experienced worker.

#### Comparison of precision

Asides from accuracy, precision is the key feature of all methods for estimating animal abundance. We used the heterogeneity parameter, α, from the negative binomial model with the smallest AIC_c_ of each of the two index methods to assess the precision of these two index methods. The variance of the negative binomial distribution is var(Y_i_) = μ_i_+α μ_i_^2^, where μ_i_ relates to the Poisson variance and α μ_i_^2^ to the extra variance. Hence, α = 0 yields the Poisson model and the more extra variance is added to the model the larger is α [37].

## Results

**Figure 2:**
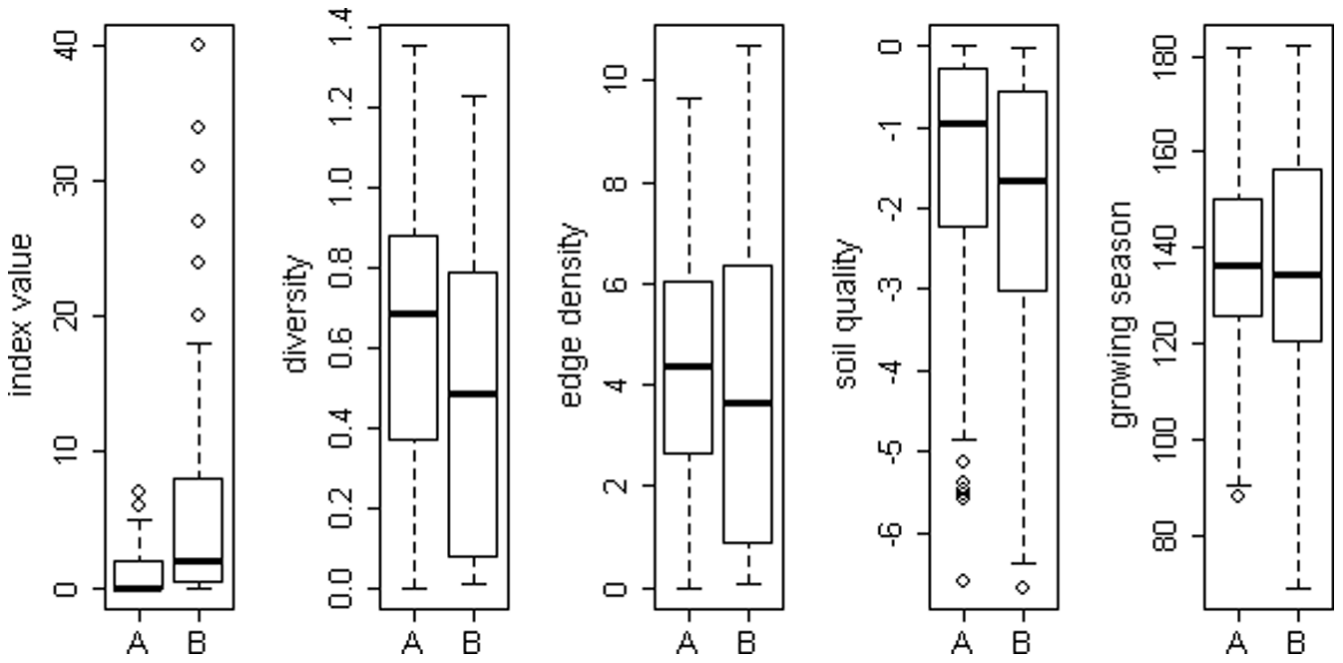
Boxplots of the distribution of the observed values of the index variable (feces count on transect, number of photographs at camera location) and the landscape variables: diversity, edge density, soil quality and growing season for feces counts (n = 262 transects, A) and camera traps (n = 88 camera locations, B).

We found between 0 and 7 feces per transect. The number of photographs at the camera locations ranged between 0 and 40 (Fig. 2). For all landscape parameters, the interquartile range (represented as the length of the box in the boxplots), a measure of dispersion, was larger in the camera trap data than in the feces count data.

The model selection process yielded seven models for the feces counts and five models for the camera traps with ΔAIC_c_<2 (compare [25] and Table S1), which we used to calculate an averaged model for each index method (Tables 1 and 2). In the model derived from the feces counts, landscape diversity and edge density had the largest relative importance (RI = 0.56) with averaged coefficients of β = 0.48 (diversity) and β = 0.061 (edge density). Whereas, in the model from the camera data, landscape diversity had more influence than edge density, where it had larger relative importance (RI = 0.65) than edge density (RI = 0.35). Also, the averaged coefficient of landscape diversity in the camera trap model (β = 0.71) was larger than in the feces counts model and the estimate for edge density was smaller (β = 0.044) than in the feces counts model. The subset averaged p-values of diversity and edge density were roughly ten times smaller in the model from camera traps than in the feces count model. Soil quality had a relative importance of RI = 0.27 and duration of the growing season had with RI = 0.19, an even lower relative importance in the feces count model. This relationship was reversed in the model from the camera traps, in which duration of the growing season had larger relative importance (RI = 0.31) than soil quality (RI = 0.14). Also, the magnitude of the averaged regression coefficients was different; the estimate of soil quality was larger in the feces count model, whereas the estimate of growing season was larger in the camera trap model. The subset averaged p-values suggested that both variables had no statistical significance in either model.

**Table 1:** Relative variable importance (RI) and full model averaged regression coefficients (averaged β) of the averaged feces count model and p-values of the subset averaged feces count model. RI indicates the sum of the weights of all models (with ΔAIC_c_<2), in which each variable was included.

	RI	Averaged β	p-value Subset Ave.
**Landscape Variables**			
Diversity	0.56	0.48	0.048
Edge Density	0.56	0.061	0.060
Soil Quality	0.27	0.027	0.26
Growing Season	0.19	7.0 e^−4^	0.48
**Confounder**			
Grass	1	0.83	0.037
Slope	1	−0.040	0.011
Visibility	1	0.23	0.81
Year 2010	1	−0.42	0.045
			

The confounder variables were not included in the selection process and hence are in all models (RI = 1).

**Table 2:** Relative variable importance (RI) and full model averaged regression coefficients (averaged β) of the averaged camera trap model and p-value of the subset averaged camera trap model. RI indicates the sum of the weights of all models (with ΔAIC_c_<2), in which each variable was included.

	RI	Averaged β	p-value Subset Ave.
**Landscape Variables**			
Diversity	0.65	0.72	0.0048
Edge Density	0.35	0.044	0.0073
Soil Quality	0.14	0.010	0.43
Growing Season	0.31	1.9 e^−3^	0.25
**Confounder**			
Vegetation	1	0.75	0.0044
Slope	1	0.011	0.48
Trail Type 2	1	0.52	0.20
Trail Type 3		1.30	0.001
Trail Type 4		1.81	<0.001

The confounder variables were not included in the selection process and hence are in all models (RI = 1).

Of the confounding variables vegetation had, as expected, a positive effect (β = 0.75) on the number of photographs, indicating more fox photographs when the proportion of ground cover was more than one third. Also, slope had, as hypothesized, a positive effect (β = 0.011), which was not statistically significant. Further, we obtained significantly more photographs on trail type 3 (hiking trail, β = 1.30) and trail type 4 (unpaved forestry road, β = 1.81) than on trail type 1 (animal path). The confounding effects of the feces counts have been described in [25].

**Figure 3:**
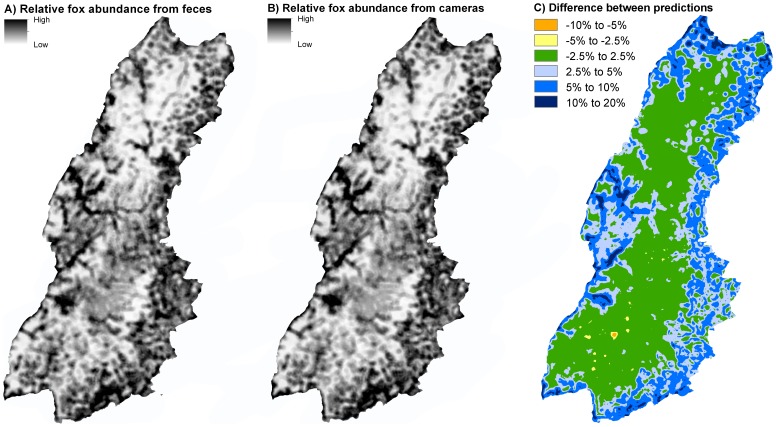
Predicted relative red fox abundance in the Black Forest extrapolated from the results of the feces counts (left) and the camera traps (middle). Difference between the two predictions in percent (prediction from feces counts – prediction from camera trap, right). The left figure is reprinted from [25] under a CC BY license, with permission from Springer, original copyright 2013.

The averaged extrapolations from the two index methods resulted in almost identical patterns of predicted relative red fox abundance in the Black Forest (Fig. 3a,b); with high predicted relative red fox abundance in the valley bottoms and the eastern Black Forest, which consist of heterogeneous landscapes.

The subtraction of the prediction from the camera traps of the prediction of the feces counts (Fig. 3c), revealed the differences between the two predictions. The differences were low with most values under 0.025 and a maximum difference of 0.16. The higher differences occurred in the areas of high predicted red fox abundance. For most locations the prediction of the feces counts was larger than the prediction of the camera traps. The correlation between the values of the two methods at 10,000 random points was 0.989 (p<0.0001).

With respect to the overall economical cost associated with performing feces counts of the 262 line transects (17057 Euros) and the number of photographs from 88 camera locations (16323 Euros) used in the analysis, the two methods were similar (Table 3). The two index methods differed significantly in the costs of equipment, which was mainly due to the initial investment, which was more than 10 times higher for the camera traps than for feces counts. The number of person days also varied significantly between the two methods, with more person days (133 days) required for the feces counts than for the camera traps (44 days).

**Table 3:** Economical costs associated with the index methods based on feces count (n = 262) and camera traps (n = 88).

	Feces counts	Camera traps
**Equipment**		
Initial costs	882 €	10864 €
Running costs equipment	0 €	200 €
Travelling costs	1536 €	939 €
**Subtotal**	**2417 €**	**12003 €**
**Labor**		
Qualified worker	61 days	18 days
Untrained worker	72 days	26 days
**Labor cost (240 € for an qualified worker per day)**	**14640 €**	**4320 €**
**Total costs**	**17057 €**	**16323 €**

The heterogeneity parameter of the top negative binomial model was smaller for the feces counts (α = 0.53) than for the camera traps (α = 0.89), meaning there was less variation in the feces counts than there was in the number of photographs.

## Discussion

We presented and used a framework that allows for comparison of two or more very different field methods of animal abundance. Further, we compared the field methods with regard to their economical costs and their precision.

In our study, both the estimated regression coefficients and the predicted relative abundance from the two index methods were very similar. There were some differences between the two methods in the magnitude of the averaged estimates of the landscape parameters, which could have been due to the close correlation of landscape diversity and edge density in the Black Forest, making a clear attribution of differences in the index to one of the two parameters impossible. The estimated regression coefficient of diversity was larger in the camera trap model than in the feces count model, whereas the regression coefficient of edge density was larger in the feces count model than in the camera trap model. The relative abundances predicted based on the parameter estimates from feces counts, versus camera traps, respectively, were almost identical. We already showed a strong correlation on the county level between the predicted relative abundance based on the feces counts and the hunting bags averaged over several years, which gave evidence that the prediction based on the feces counts is linked not only to feces abundance, but also to red fox abundance [25]. The similarity between the predictions of the two index methods added further evidence that there is a close connection between the prediction and the true relative red fox abundance in the Black Forest, and that feces counts and camera traps are both suitable methods for estimating relative red fox abundance. As we employed the camera traps in a different season and two years after the feces counts, the similarity between the predictions is even more an indicator of red fox abundance depending on landscape diversity and edge density, which both did not change between seasons and years.

We compared the two methods with regards to economic cost, precision and sample size to help wildlife ecologists choose the appropriate field technique for animal abundance estimation in their study. Regarding the economical costs, the equipment costs were by far lower for the feces counts than for the camera traps, whereas the required person days to complete the field work was higher for the feces counts. In projects with low budget, feces counts are therefore recommended if volunteers are available, whereas the high initial costs of the camera traps may pay off in long term studies.

We found differences in the precision of the two index methods; the feces count results were more precise than those based on the camera traps. The line transects used as the sampling unit for the feces counts, each cover a large cross sectional area; this increases variation within the sampling units, and reduces variation among them [38]. The camera locations, on the other hand, only capture a small area; as a consequence, the number of red fox photographs at each location varied significantly (between 0 and 47 photographs), dependent not only on differences in the landscape parameters or confounding variables, but also due to other factors that we were not able to collect, such as the proximity of camera locations to dens, resting sites, hunting grounds or travelling routes. In other studies the great variation among camera trap locations has led to reduced power to detect differences in animal abundance. [22] found significant differences for Harvey's duiker (*Cephalophus harveyi*) using line transect counts in combination with distance sampling for all but three pairwise comparisons of their six study sites, whereas using camera traps, they only found significant differences between the three most extreme observations. Further, [39] only found significant differences in the red fox picture index before and after intensive fox control in one of two years, even though the index value decreased by 23% in the non significant year. Earlier studies using track plots, which have similar statistical properties, revealed large standard deviations [40] and low power to detect significant differences [41], [42].

Even though the camera traps were less precise and the number of samples was only about one third of the sample size from the feces counts, the subset averaged p-values of landscape diversity and edge density were ten times smaller in the camera trap model than in the feces count model. We believe this was due to the fact that we selected the camera locations with prior knowledge of possible differences in relative red fox abundance and allocated more samples to extreme values of landscape diversity and edge density. This pre-selection led to the increased interquartile range in the boxplots of the two variables and by this increased the power to detect a relationship between landscape diversity and edge density and probably also increased the number of photographs. We only had this knowledge, because we already had the results of the feces counts available, which would not be the case if the studies had taken place at the same time.

The performance of camera traps as an index method can be further improved if the number of locations with zero photographs is reduced. In our study, the number of photographs could be improved by stationing camera locations only on hiking trails or unpaved forestry roads with high ground cover surrounding the camera location.

We advise wildlife ecologists to use at least two independent index methods whenever possible, to obtain more reliable estimates of abundance. Our approach can be used as framework on how to use and compare the abundance estimates from different field methods and by this enhance reliability of the abundance estimate. The approach can also be used for identifying changes in relative abundance between years or seasons, or differences between areas, simply by including a factor in the regression model and adding possible interactions between the factor and the landscape or confounder variables. The results can then be employed to obtain, for example, season specific predictions. Further, the results of different methods can also be averaged to obtain just one single prediction, but more research is needed on the practical realization of this and we refrained from doing so as our results were similar.

## Supporting Information

Table S1 **Result of the model selection of the camera trap data from the negative binomial regression model to identify variables connected with red fox abundance.** Listed are the estimated regression coefficients of the included variables, AICc, ΔAICc, and the Akaike weight of the best ranked models (ΔAICc<2).
(DOCX)
