# Supplementary material for: Toward Reliable Estimates of Abundance: Comparing Index Methods to Assess the Abundance of a Mammalian Predator
Source: PLoS One. 2014 Apr 17;9(4):e94537. doi: 10.1371/journal.pone.0094537 (PMC3990582; doi:10.1371/journal.pone.0094537)
Supplement: Table S1 — Result of the model selection of the camera trap data from the negative binomial regression model to identify variables connected with red fox abundance. Listed are the estimated regression coefficients of the included variables, AICc, ΔAICc, and the Akaike weight of the best ranked models (ΔAICc<2). (DOCX) [file pone.0094537.s001.docx]

**Supporting information**

Table­ S1: Result of the model selection of the camera trap data from the negative binomial regression model to identify variables connected with red fox abundance. Listed are the estimated regression coefficients of the included variables, AICc, ΔAICc, and the Akaike weight of the best ranked models (ΔAICc< 2).

| **Landscape variables** | | | | **Confounder variables** | | | | | |  | |  | |  | |  |
| --- | --- | --- | --- | --- | --- | --- | --- | --- | --- | --- | --- | --- | --- | --- | --- | --- |
| **diversity** | **edge density** | **soil quality** | **growing season** | | **vegetation** | **slope** | **Trail type2** | **Trail type 3** | **Trail type 4** | | **AICc** | | **delta AICc** | | **Akaike weight** | |
| 1.16 | - | - | - | | 0.76 | 0.013 | 0.52 | 1.3 | 1.8 | | 453.9 | | 0.00 | | 0.317 | |
| - | 0.13 | - | - | | 0.76 | 0.012 | 0.56 | 1.3 | 1.9 | | 454.9 | | 1.08 | | 0.185 | |
| 1.02 | - | - | 0.0055 | | 0.74 | 0.0099 | 0.46 | 1.3 | 1.7 | | 455.3 | | 1.48 | | 0.152 | |
| - | 0.12 | - | 0.0071 | | 0.75 | 0.0078 | 0.49 | 1.3 | 1.8 | | 455.7 | | 1.83 | | 0.127 | |
| 1.04 | - | 0.075 | - | | 0.76 | 0.011 | 0.59 | 1.3 | 1.9 | | 455.7 | | 1.89 | | 0.123 | |
